# Supplementary material for: Predicting habitat suitability and range shifts under projected climate change for two octocorals in the north-east Atlantic
Source: PeerJ. 2022 May 27;10:e13509. doi: 10.7717/peerj.13509 (PMC9150690; doi:10.7717/peerj.13509)
Supplement: Supplemental Information 1 [file peerj-10-13509-s001.pdf]

Predicting habitat suitability and range shifts under projected climate change for two octocorals in the north-east Atlantic

Supplementary Figures

Jenkins and Stevens 2022

Contents

Figure S1 2
Figure S2 5

Figure S1

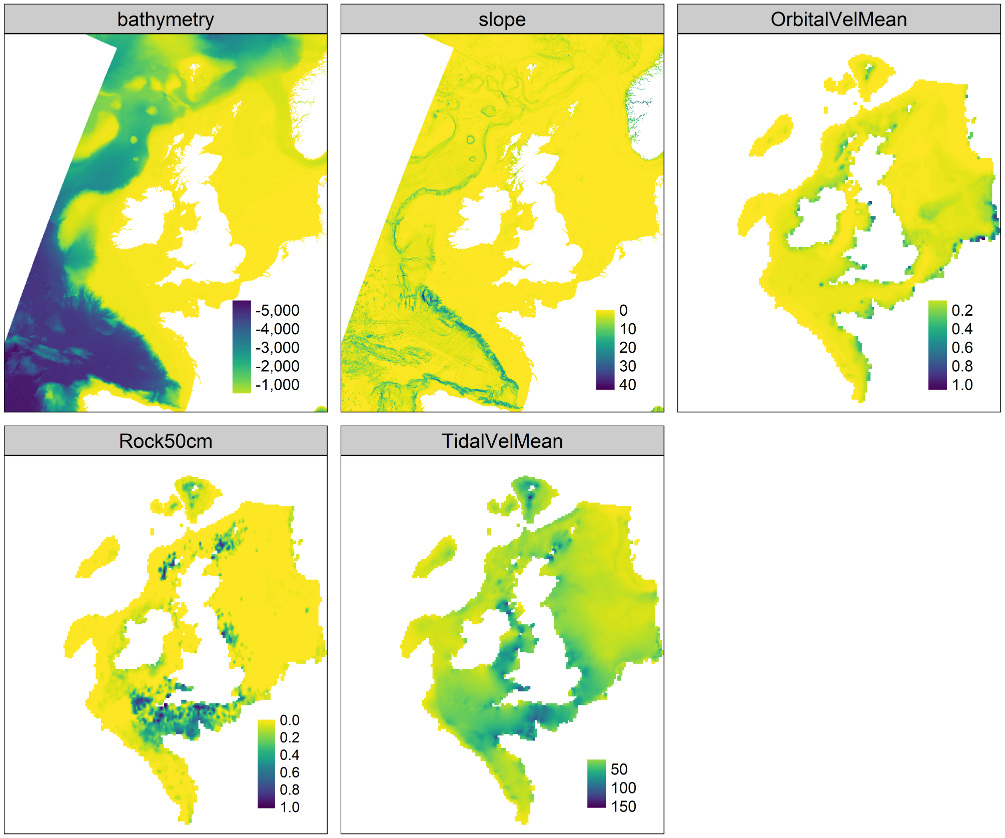

Figure S1. Thematic map of each raster predictor variable considered in the species distribution models.

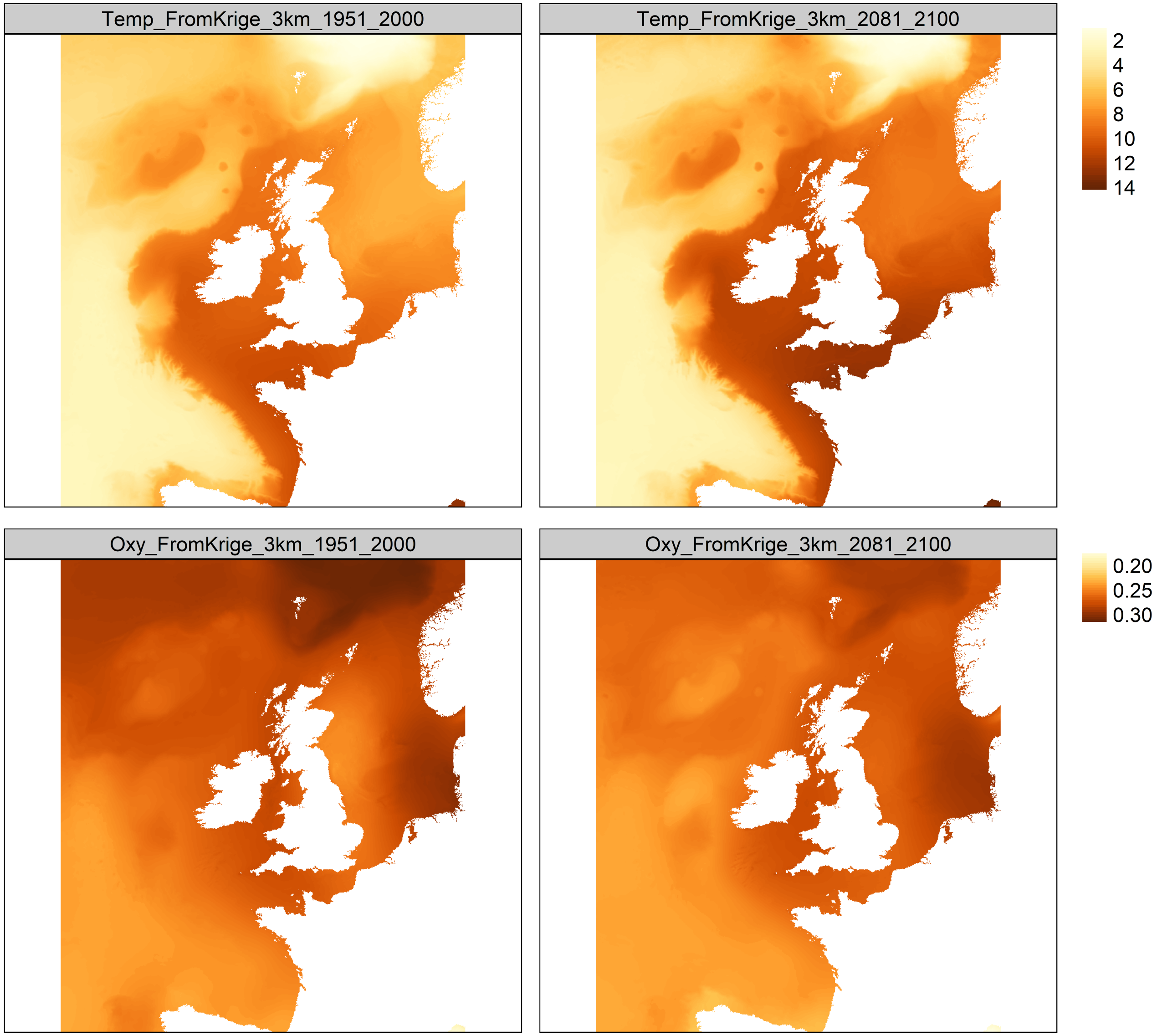

Figure S1. Thematic map of each raster predictor variable considered in the species distribution models.

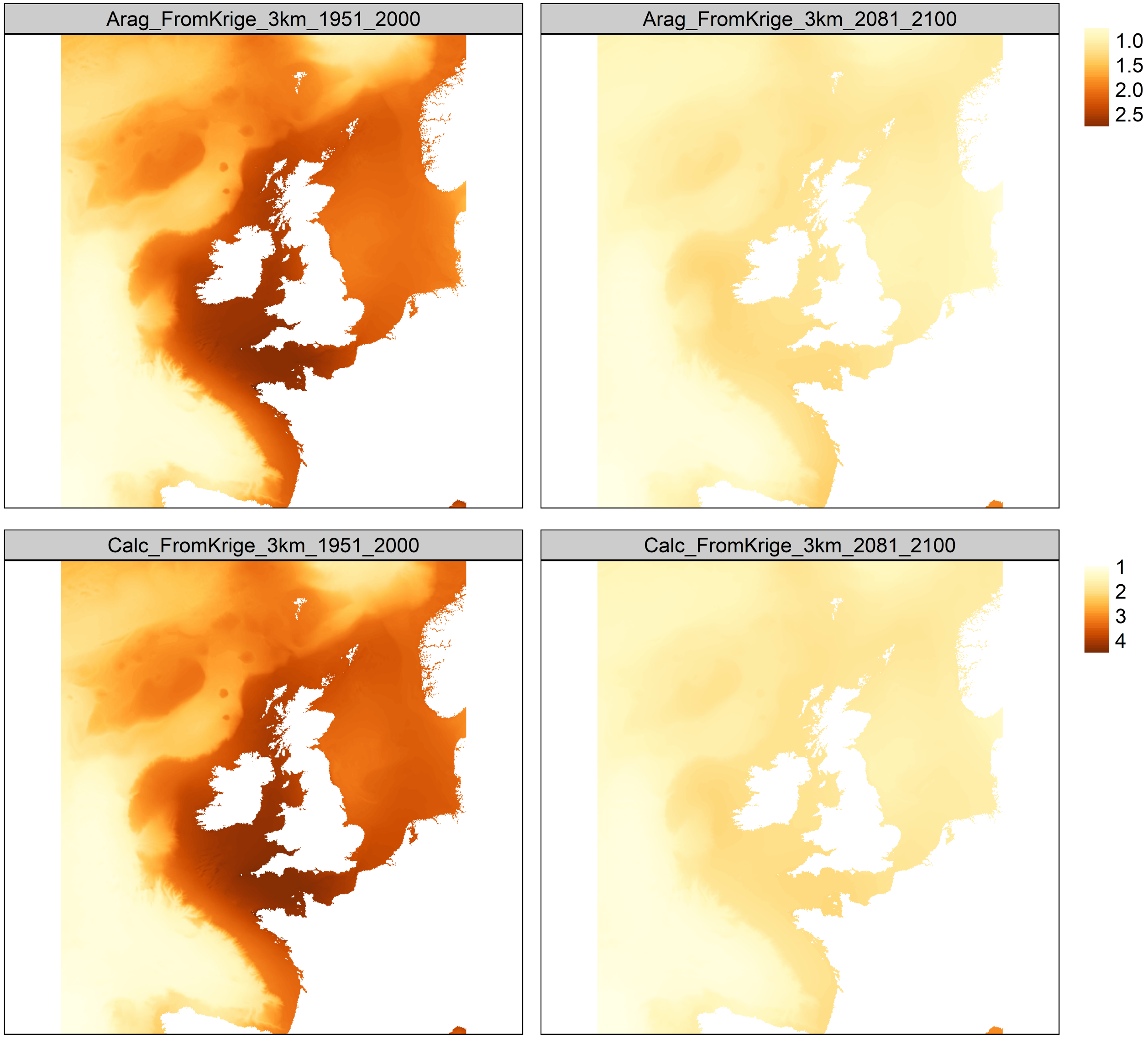

Figure S1. Thematic map of each raster predictor variable considered in the species distribution models.

Figure S2

Eunicella verrucosa

fc	rm	auc.val.avg	auc.val.sd	cbi.val.avg	cbi.val.sd	delta.AICc
L	1	0.88	0.09	0.88	0.19	1051.20
L	2	0.88	0.09	0.87	0.18	1059.57
L	3	0.88	0.09	0.88	0.18	1068.03
L	4	0.88	0.09	0.88	0.17	1076.57
L	5	0.88	0.09	0.89	0.17	1085.27
LQ	1	0.90	0.04	0.91	0.04	315.24
LQ	2	0.90	0.04	0.91	0.05	343.17
LQ	3	0.90	0.04	0.91	0.05	410.16
LQ	4	0.90	0.05	0.91	0.05	470.51
LQ	5	0.90	0.05	0.90	0.06	492.79
**H**	**1**	**0.87**	**0.09**	**0.90**	**0.08**	**0.00**
H	2	0.89	0.07	0.89	0.12	119.71
H	3	0.89	0.06	0.88	0.15	219.17
H	4	0.89	0.06	0.90	0.13	340.21
H	5	0.89	0.06	0.88	0.15	326.24

Alcyonium digitatum

fc	rm	auc.val.avg	auc.val.sd	cbi.val.avg	cbi.val.sd	delta.AICc
L	1	0.76	0.13	0.71	0.33	3436.31
L	2	0.76	0.13	0.71	0.32	3269.81
L	3	0.76	0.13	0.71	0.32	3129.00
L	4	0.76	0.13	0.71	0.32	3026.15
L	5	0.76	0.13	0.70	0.33	2927.29
LQ	1	0.78	0.10	0.95	0.06	562.41
LQ	2	0.78	0.11	0.96	0.03	980.09
LQ	3	0.77	0.11	0.93	0.09	1398.29
LQ	4	0.77	0.12	0.87	0.15	1825.30
LQ	5	0.77	0.13	0.78	0.25	2311.19
H	1	0.79	0.10	0.95	0.05	0.93
**H**	**2**	**0.80**	**0.09**	**0.95**	**0.05**	**0.00**
H	3	0.80	0.09	0.96	0.06	34.15
H	4	0.80	0.09	0.96	0.06	39.58
H	5	0.80	0.08	0.96	0.06	108.69

Figure S2. Results of model evaluation for the pink sea fan (*Eunicella verrucosa*) and dead man's fingers (*Alcyonium digitatum*).
